# The Role of Curcumin in Oral Health and Diseases: A Systematic Review

**DOI:** 10.3390/antiox13060660

**Published:** 2024-05-28

**Authors:** Francesco Inchingolo, Alessio Danilo Inchingolo, Giulia Latini, Irma Trilli, Laura Ferrante, Paola Nardelli, Giuseppina Malcangi, Angelo Michele Inchingolo, Antonio Mancini, Andrea Palermo, Gianna Dipalma

**Affiliations:** 1 Department of Interdisciplinary Medicine, University of Bari “Aldo Moro”, 70124 Bari, Italy or a.inchingolo1@studenti.uniba.it (A.D.I.); or giulia.latini@uniba.it (G.L.); or irma.trilli@uniba.it (I.T.); or laura.ferrante@uniba.it (L.F.); drnardellipaola@gmail.com (P.N.); or a.inchingolo3@studenti.uniba.it (A.M.I.); or antonio.mancini@uniba.it (A.M.); or gianna.dipalma@uniba.it (G.D.); 2 College of Medicine and Dentistry, Birmingham B4 6BN, UK; andrea.palermo2004@libero.it

**Keywords:** curcumin, antioxidants, natural products, oral health, oral diseases, polyphenols

## Abstract

Curcumin (Curcumin) belongs to the polyphenol family. It is extracted by drying the root of a plant of Asian origin, belonging to the *Zingiberaceae* family. The best-known species is *Curcumincuma Longa*. Curcumin has been recognized as having great therapeutic powers since ancient times. Studies on curcumin have since confirmed its powerful antioxidant properties, preventing both the formation of free radicals and their neutralization, having anti-inflammatory, antibacterial, immunological, and neuroprotective properties, as well as being a regulator of the intestinal microbiota with beneficial effects on the clinical manifestations of metabolic syndrome. Our study aimed to highlight how all these therapeutic aspects could benefit oral health, both preventing and improving the course of pathological processes. The effect of mouthwashes, and curcumin-based gels on the regulation of bacterial plaque and in the control of gingivitis, was largely comparable to that of using 0.20% chlorhexidine, with fewer side effects. Being a highly hydrophobic substance, it has a high permeability to cross the cell membrane. Bioavailability increases when combined with liposoluble substances (e.g., olive oil) and piperine, which improves absorption. Curcumin also has a negligible degree of toxicity, making it an excellent alternative to the use of gold standard products for oral disinfection.

## 1. Introduction

**Figure 1:**
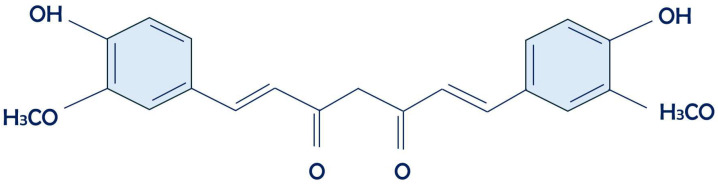
Curcumin’s chemical structure.

Curcumin (*diferuloylmethane*) (Figure 1) is one of the most studied polyphenols in recent years [1,2,3]. It belongs to the Zingiberaceae family and derives from the drying of the root of a plant of Asian origin: Curcumincuma longa. Since ancient times, Curcumincuma has been recognized for its multiple therapeutic properties, which have been confirmed by recent clinical studies [4,5,6].

Recent scientific research on curcumin has demonstrated that it possesses the following properties: anti-inflammatory, antioxidant, antifungal, antidepressant, healing (therapeutic in Crohn’s disease, ulcerative colitis, and peptic and gastric ulcers), and as an adjuvant in acute coronary syndrome therapies, chemotherapies, and as a hypoglycemic [7]. Curcumin also stands out for its ability to positively influence intestinal microbiota, thereby contributing to maintaining an optimal microbial balance. This effect can have significant implications in the prevention and treatment of gastrointestinal disorders, thereby enhancing overall health [8,9,10]. Additionally, recent studies have highlighted Curcumin has potential in supporting brain health and cognitive function, opening intriguing avenues in the treatment of neurodegenerative disorders, such as Alzheimer’s and Parkinson’s diseases [11,12].

Research continues to explore new applications and potential synergies of curcumin with other natural substances and conventional drugs, aiming to maximize its therapeutic benefits [13,14,15]. Furthermore, interest in its anti-inflammatory and antioxidant properties extends beyond the medical sphere, with potential applications in the food and cosmetic industries. Curcumin also protects against risk factors such as smoking, alcohol, and stress [16,17,18].

The ascertained pleiotropic nature of curcumin creates interference in complex biological processes and various inflammatory factors that regulate oxidation–reduction processes, such as reactive oxygen species (ROS), cytokines, cyclooxygenase-2 (COX-2), interleukins (ILs), nuclear factor kappa B (NF-κB), C-reactive proteins, transforming growth factor-β (TGF-β), and other enzymes involved in inflammation [19,20,21]. Curcumin inhibits vascular endothelial growth factor (VEGF) and various kinases, demonstrating a controlling action in angiogenesis and the growth of cancerous lesions [22,23].

**Figure 2:**
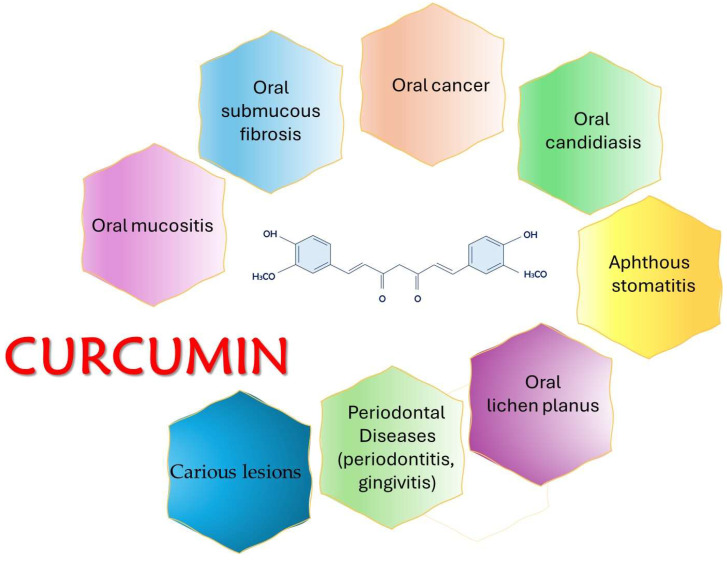
Curcumin’s importance in different clinical situations.

Due to its medicinal and pharmacological properties, curcumin has also found application in the treatment of multiple diseases of the oral cavity (caries, periodontitis, gingivitis, aphthous stomatitis, oral candidiasis, mucositis, oral submucosal fibrosis, lichen planus, oral leukoplakia, carcinogenic lesions, etc.) (Figure 2), which affect approximately 3.5 billion people worldwide [24,25,26].

Classic therapeutic protocols can often cause side effects, such as resistance to antibiotics, the alteration of the microbiota, vomiting, diarrhea, and pigmentation [27,28,29].

Due to its poor oral bioavailability, non-hydrophilic nature, and rapid degradation, curcumin’s therapeutic potential is limited. With oral administration, approximately 40–85% of curcumin passes through the gastrointestinal tract unabsorbed [30,31]. It is largely metabolized in the intestine and liver and rapidly eliminated. Curcumin may interfere with hypoglycemic drugs [32].

Different formulations of use have been studied (tablets, liposomal encapsulation, emulsions, capsules, nanoparticles, gels, ointments, and powder) to improve their bioavailability [30]. It has been administered alone or combined with other substances (lycopene and piperine, honey, green tea, tulsi, triamcinolone-hyaluronidase gel, and photodynamic therapy) [33]. In addition to being effective, it is safe (safety dosage of 12 g/day for 3 months) and well tolerated in patients with oral pathologies [34,35,36]. During dental caries prophylaxis, the bio-nanocomposite carboxymethyl starch (CMS)–chitosan (CS)–montmorillonite (MMT) was developed to better trap curcumin (91%) and allow its slow release. This formulation was particularly effective against *Streptococcus mutans* as well as significantly preventing and slowing down the formation of biofilms on dental elements [37].

Curcumin-containing toothpastes have been produced, recognizing their effectiveness in preventing dental plaque and gingivitis due to their antimicrobial action [30].

Research has established the therapeutic action of curcumin as an adjuvant in the treatment of periodontal diseases (gingivitis, periodontitis, carious lesions, and loss of alveolar bone). The use of curcumin gel or curcumin nanoparticles has shown a positive effect on clinical, symptomatic, and microbiological aspects in addition to scaling and root planing (SRP) [38].

The periodontal index, including plaque index (PI), gingival index (GI), and periodontal pocket depth (PPD), and bleeding on probing (BOP), was improved, as was the level of salivary procalcitonin (PCT), the receptor activator of nuclear factor κB (RANK), and the receptor activator of nuclear factor κB ligand (RANKL), resulting in less alveolar bone loss [39,40,41].

Studies on the use of mouthwash containing 1% curcumin have shown overlapping anti-plaque and anti-gingivitis properties compared to a 0.2% chlorhexidine gluconate mouthwash; therefore, they can be integrated with SRP. The use of a 2% curcumin gel, with a higher pharmacological activity, can be used as a supplement in the therapies and prophylaxis of periodontal pockets [42,43,44].

The use of curcumin has also been evaluated in oral submucosal fibrosis (OSMF), a chronic fibrotic disease of the mouth, oropharynx, and upper part of the esophagus, prevalent in males between 20 and 40 years of age, progressively causing serious functional problems and predisposing to the occurrence of malignant carcinogenic lesions [45,46,47].

The administration of curcumin, both systemically (tablets) and topically (ointment), has shown a significant reduction in the clinical state and symptoms with a reduction in burning sensation and an increase in the mobility of the tongue and the opening of the lips and cheeks. A reduced presence of the proteins responsible for OSMF (p53, TGF-β, and iNOS) was recorded after curcumin therapy [48,49].

Research on Curcumin use in mucositis reported interesting results on the use of curcumin in a pathology that occurs after chemotherapy and radiation treatments, and it is well tolerated in pediatric oncology patients [50,51].

Its inflammatory effects are expressed in the reduction in the inflammatory cytokines IL-6 and IL-8. Furthermore, the reduction in fibrotic cicatricial function is expressed by inhibiting fibroblasts and myoblasts (type I and III collagen) of the oral mucosa [52]. Furthermore, curcumin associated with capsaicin induces apoptosis, has photosensitizing properties in the presence of oxygen and sunshine, and therefore can be used in photodynamic therapy, especially in precancerous oral diseases [53,54,55].

The levels of vitamins C and E, antioxidant substances, are increased with curcumin treatment, preventing lipid peroxidation, the production of free radicals, and therefore cellular damage [56]. Curcumin studies on oral cavity tumors are interesting, especially oral squamous cell carcinoma (OSCC), the most frequent (90%) of oral cavity tumors and the sixth in the ranking of tumors, which has a high mortality potential [57,58].

Research found that curcumin therapy in OSCC reduced the metalloproteinases MMP-2 and MMP-9 and markers of epithelial–mesenchymal transition (EMT): Snail, Twist, and E-cadherin, their expression marking the invasiveness of oral cancer. It stimulated tumor protein 53 (p53), a cell cycle transcription factor, which acts as a tumor suppressor in EMT cases [59,60,61,62,63].

In the chewing gum formulation, the serum values of the biomarkers CXCL1 (GRO-α) and TNF-α, after chewing the gum and allowing it to act on the mucous membranes, were significantly decreased [64,65].

Overall, studies have found a marked improvement in the antitumor immune response after the treatment of OSCC with curcumin by both apoptosis and autophagy [66,67,68]. There was an increase in CD8 + T cells, also called cytotoxic T lymphocytes, capable of inducing cellular apoptosis, and a decrease in Treg (suppressor T cells) and myeloid-derived suppressor cells (MDSCs), as well as the presence of autophagic vacuoles [69,70,71].

Every finding from the most current research indicates that using curcumin for the treatment of the various expressions of oral pathologies can offer the possibility of valid therapies and prophylaxis [72,73]. Further research, however, is necessary to understand the real therapeutic potential of curcumin, defining the optimal administration methods and the right dosages.

Research continues to explore new applications and potential synergies of curcumin with other natural substances and conventional drugs, aiming to maximize its therapeutic benefits. Furthermore, interest in its anti-inflammatory and antioxidant properties extends beyond the medical sphere, with potential applications in the food and cosmetic industries [34].

Ultimately, curcumin represents a significant natural compound with a broad spectrum of biological and therapeutic activities. Ongoing research and development could lead to new discoveries and opportunities in the field of medicine and human well-being.

Our study highlights how all these therapeutic aspects could benefit oral health, both preventing and improving the course of pathological processes [74].

## 2. Materials and Methods

In this paper, we conducted a systematic review following the Preferred Reporting Items for Systematic Reviews and Meta-Analyses (PRISMA) guidelines using the PubMed, Web of Sciences (WOS), and Scopus databases with the PROSPERO code ID CDR 536228. We used the following keywords: “Curcumin” AND “oral disease” AND “oral health” with articles from the last 10 years searched, favoring the topic related to the effects of curcumin in healthy and disease conditions [75]. The included studies contained information about the use of curcumin in vivo and were all full-text articles. Furthermore, all papers reporting in vitro experiments, animal models, as well as literature reviews with/without meta-analyses were excluded. Articles written in English were selected, and the final search was conducted on 18 March 2024. The references of the included literature were thoroughly examined to identify additional papers that could be relevant.

Two authors (I.T. and G.L.) performed the article extraction according to the inclusion criteria, and any disagreement was resolved after adequate discussion and comparison.

## 3. Results

**Figure 3:**
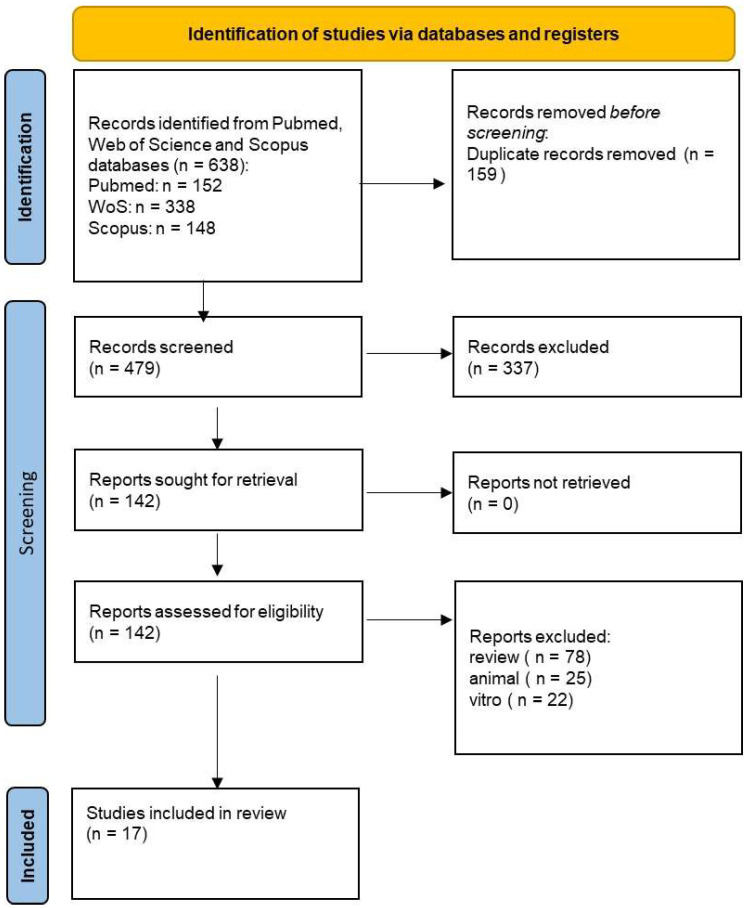
PRISMA flow-chart following the guidelines.

Initially, a total of 638 documents were identified through the literature search: 152 from PubMed, 338 from Web of Science (WOS), and 148 from Scopus. After removing 159 duplicate articles, 479 articles were assessed. Subsequently, we screened for eligibility and inclusion criteria, resulting in the inclusion of 17 papers. The study process and PRISMA flowchart are summarized in Figure 3, while detailed summaries of the included articles can be found in Table 1.

### Quality Assessment and Risk of Bias of the Included Articles

**Figure 4:**
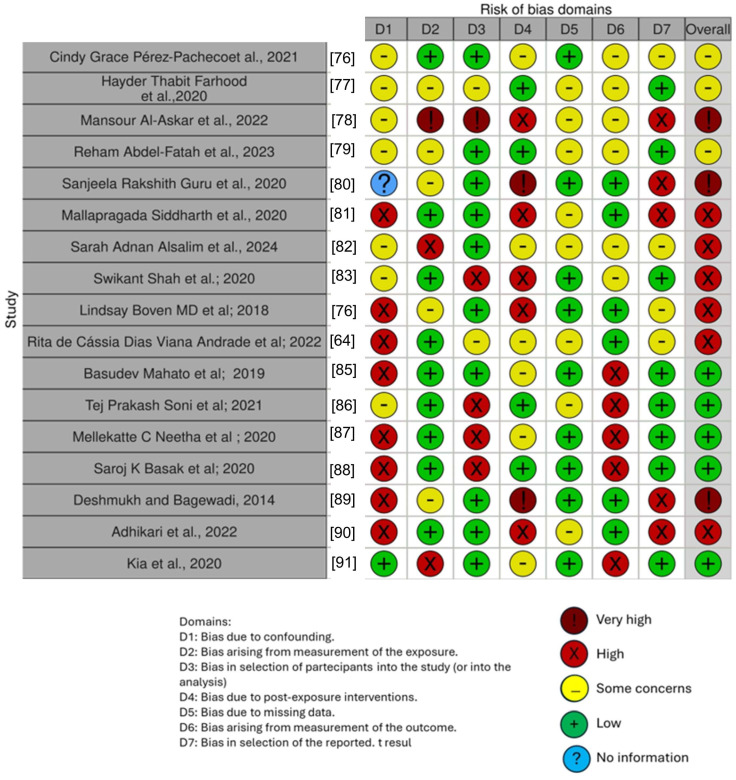
Bias assessment [76,77,78,79,80,81,82,83,85,86,87,88,89,90,91].

The risk of bias of the included studies is reported in Figure 4. Regarding the bias due to confounding, most studies had a high risk. The bias arising from measurements was a parameter with a low risk of bias. Many studies had a low risk of bias due to bias in the selection of participants. Bias due to post-exposure could not be calculated due to high heterogeneity. Bias due to missing data was low in many studies. Bias arising from the measurement of the outcome was low. Bias in the selection of the reported results was high in most studies. The final results show that five studies had a low risk of bias, three had a very high risk of bias, and six had a high risk of bias.

## 4. Discussion

### 4.1. Curcumin and Periodontology

Periodontitis, an inflammatory condition caused by periodontal bacteria, leads to tooth loss, alveolar bone loss, and gradual tissue damage. The mechanical removal of bacterial biofilms, such as through SRP, is a crucial aspect of periodontal therapy [92]. However, deep periodontal pockets and intricate root furcations are often difficult to access. Therefore, antimicrobial drugs or antibacterial substances are frequently used in conjunction with mechanical debridement to enhance therapy effectiveness [93,94,95].

The study by Hayder Thabit Farhood and Basma Ghafori Ali investigated a curcumin oral gel as an adjunct to subgingival SRP for managing 5–7 mm periodontal pockets. Twenty patients with periodontitis participated in the split-mouth intervention. The results showed significant improvements in the clinical parameters over one month in both the test and control groups, including PI, GI, PPD (periodontal probing depth), and relative CAL (clinical attachment level). BOP significantly decreased in the test group, indicating curcumin’s anti-inflammatory effect. While both groups showed similar improvements, the test group exhibited a more pronounced reduction in bleeding on probing. The study concluded that curcumin demonstrates promise as an adjunctive therapeutic agent in periodontal treatment, particularly in improving clinical parameters and reducing inflammation [77,96].

The purpose of Reham Abdel-Fatah’s research was to assess the efficacy of a curcumin gel as a local medication delivery method for treating periodontitis, focusing on its impact on the clinical parameters and PCT levels. The study involved 54 participants, including patients with periodontitis and healthy volunteers who underwent SRP followed by the application of the curcumin gel or no additional treatment [79].

(PI, GI, and PPD) and CAL were considered, and salivary samples were collected to analyze PCT levels before and after the treatment. The results demonstrated that the group that received the curcumin gel showed greater improvements in PI, PPD, and CAL than the group that received SRP alone [97].

Salivary PCT levels also decreased significantly after the treatment. These results suggest that the application of the curcumin gel in addition to SRP can effectively improve periodontal health and reduce inflammatory biomarkers, such as salivary PCT [79].

Cindy Grace Pérez-Pacheco’s study investigated the efficacy of adding nanoencapsulated curcumin to non-STP. Participants diagnosed with generalized periodontitis were recruited based on specific criteria and the protocol involved applying curcumin-loaded nanoparticles to selected periodontal sites after SRP. Clinical parameters, inflammatory cytokine levels, and subgingival microbiota were evaluated for six months. The results showed significant improvements in the clinical parameters in both the test and control groups, with slightly better results observed in the curcumin-treated sites. Changes in cytokine expression and subgingival microbial composition were also noted, suggesting potential benefits of adjunctive curcumin therapy. However, the study found that a single application of nonencapsulated curcumin did not significantly improve the treatment outcomes and recommended further research to explore the effects of repeated applications and different concentrations of curcumin [76].

The study by Al-Askar et al. aimed to compare curcumin analgesic efficacy with mefenamic acid (MA) following a surgical periodontal treatment [78]. Two groups of seventy-six periodontitis patients were created, one receiving curcumin and the other MA. Both groups received postoperative antibiotic therapy. Pain was assessed using numeric and verbal rating scales at 24, 48, and 72 h. No significant differences were observed in pain scores between the two groups. Curcumin was found to be ineffective in managing pain compared to MA. However, it was noted that the lack of operator blinding may have influenced the results [78].

The study by Sanjeela Rakshith Guru investigated curcumin and chlorhexidine’s effectiveness as local drug delivery agents for treating chronic periodontitis. Patients aged between 25 and 50 years with mild-to-moderate periodontitis were selected. They received either a 2% curcumin nanogel or 1% chlorhexidine gel alongside SRP. Clinical parameters, such as PI, GI, probing pocket depth, and CAL, were assessed at baseline, 21 days, and 45 days. The microbiological analysis targeted periodontal pathogens, including *Aggregatibacter actinomycetemcomitans*, *Porphyromonas gingivalis*, and *Tannerella forsythia*. Both treatments significantly improved the clinical parameters, with comparable antibacterial effects against periodontal pathogens. The curcumin nanogel exhibited potential as an (LDD) agent, warranting further investigation with larger sample sizes and longer follow-up periods [80].

The composition and effectiveness of a 2% curcumin gel as a local medication delivery agent in a chronic periodontitis treatment were examined in the trial conducted by Mallapragada Siddharth, comparing its effects with 0.2% chlorhexidine gel. Patients with chronic periodontitis were recruited, and the periodontal parameters were assessed at baseline, 1 month, and 3 months after treatment. A microbiological analysis was also conducted. The results showed a significant reduction in periodontal parameters and microbial counts in both the experimental and control groups, with a higher reduction observed in the curcumin gel group. Curcumin demonstrated anti-inflammatory and antibacterial properties, making it a promising adjunct to SRP in periodontal therapy. More research with longer follow-up times and larger sample sizes is warranted to explore its full potential in periodontal treatment [81].

The examined studies support curcumin’s potential as a therapeutic adjuvant for the treatment of periodontitis. The research highlighted significant improvements in clinical and microbiological parameters in patients treated with curcumin, both as an oral gel and as a nano-formulation, in addition to SRP. The effectiveness of curcumin in reducing inflammation and improving periodontal health has been demonstrated through reductions in PI, GI, PPD, and CAL. Furthermore, significant reductions in bleeding on probing and PCT levels have been observed, indicating an anti-inflammatory action. However, further research is required to explore more widely the therapeutic potential of curcumin and define optimal dosages and administration modalities. The results obtained to date suggest that curcumin represents an important resource in the management of periodontal health and oral diseases.

### 4.2. Curcumin and Oral Mucositis

Oral mucositis is a recurrent side effect of chemoradiation treatment for head and neck cancers (HNCs), characterized by inflammation and ulceration of mouth and throat mucous membranes [98]. This condition can impair nutrition, speech, and swallowing, negatively affecting the patients’ quality of life. Management strategies focus on pain relief, infection prevention, and supportive care to promote the healing of oral tissues [99,100].

Recent studies suggest that curcumin may play a significant role in the treatment of oral mucositis due to its anti-inflammatory, antioxidant, and healing properties [101,102].

In this regard, Sarah Adnan Alsalim’s study assessed the use of a curcumin-based oral gel in comparison to the magic solution (dexamethasone mouthwash), as a standard treatment for radiation-induced oral mucositis (RIOM). It demonstrated a significant reduction in the severity of oral mucositis and increased levels of salivary EGF. Significant variations were observed between the groups in terms of the VAS score and the WHO scale.

These results imply that the gel might indeed be a useful tool for managing and preventing RIOM [83].

In Tej Prakash Soni’s study, the bio-enhanced turmeric formulation (BTF) used consisted of a mixture of curcuminoids, mainly curcumin and turmeric essential oil, showing a significant reduction in severe oral mucositis, dysphagia, oral pain, and dermatitis in patients with oral cancer undergoing chemotherapy. Sixty patients who underwent radical surgery were randomized into three groups: groups A and B received bio-enhanced curcumin capsules at a low dose (1 g/day) or a high dose (1.5 g/day), respectively, and group C received the placebo. The treatment was administered daily for 6 weeks in conjunction with chemoradiotherapy.

The evaluated endpoints highlighted how the BTF can significantly reduce severe oral mucositis induced by chemoradiotherapy, dysphagia, oral pain, and dermatitis in patients with oral cancer [86].

Additionally, Rita de Cássia Dias Viana Andrade’s study looked at the impact of antimicrobial photodynamic therapy (aPDT), which was mediated by curcumin and photobiomodulation (PBM), as an adjuvant treatment for oral mucositis in cancer patients receiving chemotherapy and/or radiation therapy. The results showed that the aPDT group experienced early clinical improvement and a significant reduction in the degree of mucositis and associated pain when compared to the control group (nystatin-treated group). Regarding the antibacterial effect, aPDT showed a higher decrease in Candida yeasts in the parameters that were examined [84].

Lastly, a study comparing the efficacy of a 0.15% benzidamine-based mouthwash and curcumin mouthwash on RIOM patients discovered that, while neither mouthwash was completely successful in delaying the onset of RIOM or lessening its severity, using a 0.1% curcumin mouthwash was able to achieve this [82].

As a result, treating oral mucositis is a considerable problem for the care of patients receiving chemoradiation therapy for head and neck cancer. The aforementioned studies demonstrate how curcumin, whether in the form of an oral gel, formulations like BTF (BCM-95^®^), or curcumin -mediated antimicrobial photodynamic therapy (aPDT), can be considered an appropriate treatment for the management of oral mucositis. These formulations have demonstrated notable advantages in terms of symptom severity reduction and tissue healing [103,104].

### 4.3. Curcumin and Potentially Malignant Disorders

Potentially malignant disorders are a category of pathological conditions characterized by tissues having certain signs of abnormality, but not yet clearly cancerous. Their potential to develop malignant tumors requires careful evaluation and monitoring.

In this regard, it has been studied the use of natural substances, easy to find and with low toxicity, such as curcumin or green tea, for the possibility of being able to reverse, stabilize, or stop the progression of such oral potential malignant disorders (OPMDs), as evaluated in the randomized controlled trial of Mellekatte C Neetha. Specifically, to evaluate the effectiveness of such substances, the biomarkers (Ki67, cyclin D1, and p53) of the 60 patients with OPMD, divided into three groups of 20, and randomized to receive for 3 months a green tea extract [topical + systemic (800 mg/day)] or Curcumin [topical + systemic (950 mg/day)] or combined therapy, were analyzed. It was shown that the combined effect of these substances can be used as an alternative in oral cancer chemoprevention, downregulating molecular biomarkers in a short time [87].

The study by Basudev Mahato and colleagues assessed the efficacy of a commercially available combination called BIOCUMIN, which contains piperine (5 mg), lycopene (25 mg), and curcumin (500 mg), twice daily for three months, on 40 patients with a clinical and histopathological diagnosis of OSMF. The visual analog scale (VAS) was used to measure and assess the clinical parameters before and after treatment, including burning sensation, mouth opening (MO), mucosal flexibility (MF), and tongue protrusion (TP).

Both the burning feeling and the VAS score improved at the end of the course of treatment. Furthermore, there was an improvement in MF and TP along with a rise in MO.

The histopathological investigation revealed a significant decrease in collagen deposition and an increase in epithelial thickness, indicating re-epithelialization, as observed at the end of treatment by immunohistochemistry tests using Col1A1.

Thus, using this combination of medications has often shown to be an effective and practical way to manage OSMF [85,105].

In summary, disorders that have the potential to become malignant necessitate careful monitoring and a precise evaluation to estimate the possibility that they will develop into malignant tumors. Studies conducted recently have demonstrated the ability of natural compounds, such as curcumin and green tea, to lower the chance of precancerous lesions progressing. Curcumin, lycopene, and piperine together have shown promise in treating precancerous diseases, offering chemopreventive options for oral cancer and enhancing the quality of life of patients.

### 4.4. Curcumin and Oral Cancer

Oral cavity cancer is one of the most common malignant tumors that can severely affect the health and quality of life of patients.

The standard treatment includes surgery, radiotherapy, and chemotherapy, but the prognosis is often difficult, especially in the advanced stages of the disease.

For this reason, interest in alternative and adjuvant therapeutic approaches, such as the use of curcumin, is increasing significantly in research, with the aim to improve treatment effectiveness and the survival of oral cancer patients [106,107,108].

Curcumin, due to its anticancer properties, was identified as a substance for the chemoprevention of head and neck squamous cell carcinoma, but its use was limited due to its poor bioavailability [109,110].

In this regard, the study conducted by Lindsay Boven et al. in 2018 evaluated the effectiveness of a new gum formulation to improve direct absorption from the mucosa to the bloodstream. By using two different models of chewing, the initial chewing protocol (30 min gum chewing) and the revised chewing protocol (alternating between gum chewing and 30 min placement of gum against the buccal mucosa), it was shown that an increased contact with the mucosa turns out to be fundamental in order to improve the release and the absorption of the curcumin [64].

Another investigation by Saroj K. Basak assessed the efficacy of a botanical medication known as APG-157, which contains curcumin and other polyphenols, in the management of oral cancer. The analysis of the salivary microbial flora in 13 healthy subjects and 12 oral cancer patients revealed a decrease in *Bacteroidetes* species in cancer patients, while RNA and the immunofluorescence analysis of cancer tissues revealed an increase in immune T cells in the tumor microenvironment and a release of inflammatory cytokines (IL-1β, IL-6, and IL-8) in cancer patients [111].

According to these findings, APG-157 may find application as a medicinal medication in conjunction with immunotherapy [88].

Therefore, the use of curcumin, showing encouraging results, presents itself as a promising prospect in the treatment of oral cancer. These studies provide a substantiated basis for further research and for the development of effective adjuvant therapies, paving the way for more targeted and less invasive potential therapeutic approaches.

### 4.5. Curcumin and Other Clinical Conditions

The research on the effect of curcumin on precancerous lesions of the mouth, such as leukoplakia, oral submucosal fibrosis, and lichen planus, by Balwant Rai et al. in 2010 involved patients with some pathologies, including precancerous lesions, and healthy individuals. The study evaluated the salivary and serum markers of oxidative stress, including malondialdehyde (MDA) and 8-hydroxydeoxyguanosine (8-OHdG), and vitamins C and E before and after taking curcumin. The results showed increased levels of vitamins C and E and decreased MDA and 8-OHdG in patients with precancerous lesions after taking curcumin, with a significant improvement in lesion size and pain. Furthermore, curcumin increased the ability to open the mouth in patients with submucosal fibrosis. The effects were particularly evident after the clinical healing of the lesions, suggesting that curcumin exerts its anti-precarcinogenic activities by elevating the levels of vitamins C and E and preventing lipid peroxidation and deoxyribonucleic acid (DNA) damage [112]. In 2014, Radha A. Deshmukh and Anjana S. Bagewadi conducted a randomized clinical trial to assess the efficacy of a curcumin versus a triamcinolone acetonide gel in treating minor RAS. Sixty RAS patients were randomly assigned to either the curcumin gel treatment or the triamcinolone acetonide gel treatment groups. After applying the gel three times daily to their ulcers, both groups’ ulcer sizes, numbers, and duration of pain reduction were evaluated. Within 7 days, both treatments resulted in a significant reduction in pain, size, number, and duration of ulcers, with no observable difference in efficacy between the two groups. A statistical analysis was conducted using the Mann–Whitney U test [89].

In Adhikari’s 2022 study, turmeric, known for its anti-inflammatory, antioxidant, and fibrinolytic properties, was shown to have anti-tumor capabilities, reducing the activity of inflammatory and cell growth factors associated with precancerous and cancerous conditions. This polyphenolic compound has demonstrated beneficial properties in the treatment of oral pathologies such as OSMF, a chronic and progressive disease that can lead to malignant transformation. Thirty-four patients participated in this randomized clinical trial, which assessed the effectiveness of curcumin in treating OSMF when combined with intralesional dexamethasone and hyaluronidase. Compared to the placebo-only control group, the patients treated with curcumin showed a substantial improvement in MO, cheek flexibility, and TP. The findings suggest that the combination of curcumin, dexamethasone, and hyaluronidase may be an effective treatment for OSMF, but larger studies are needed to confirm these findings and evaluate the effectiveness of the treatment across all stages of the disease [90].

In 2012, Chainani-Wu et al. investigated the efficacy of curcumin, specifically a standardized extract called “Curcumin C3 complex,” in managing OLP, a condition characterized by inflammation in the mouth [113]. OLP is a chronic mucosal disease identified as an immune disorder, presenting various lesion forms, including reticular, papular, plaque-like, bullous, erosive, and ulcerative. The prevalence of OLP is estimated to be between 0.55% and 2%. The curcuminoids were provided in tablet form along with identical placebo tablets. The study involved randomization and blinding to ensure unbiased results. The measurement of the response variables included assessing symptom intensity using the numerical rating scale (NRS), evaluating clinical signs using the modified oral mucositis index (MOMI), monitoring side effects, and measuring levels of C-reactive protein (CRP) and interleukin-6 (IL-6) in the blood. The results showed that curcumin significantly reduced the symptoms and signs of OLP compared to the placebo group. The adverse effects were minimal and similar between the curcuminoids and placebo groups. However, changes in CRP and IL-6 levels were not statistically significant between the groups. The correlation analyses suggested potential associations between baseline CRP/IL-6 levels and OLP severity, as well as changes in CRP/IL-6 levels and treatment responses, although these associations did not reach statistical significance. The study concluded that high-dose curcuminoids effectively reduced the symptoms and signs of OLP and were well-tolerated. Future research should explore the role of CRP and IL-6 in OLP and the mechanism of action of curcuminoids in treating inflammatory conditions [113]. While OLP lesions commonly appear on the buccal mucosa, they can also affect other oral cavity areas. OLP presentation is primarily symptom-related, ranging from a burning sensation to severe pain, rarely remitting spontaneously. Management focuses on symptom reduction and monitoring for dysplastic changes. Corticosteroids are the first-line treatment but can cause unwanted side effects, prompting the search for alternatives like curcumin, known for its antioxidant, anti-inflammatory, antimicrobial, and anticancer properties. However, curcumin faces bioavailability limitations. A first-of-its-kind study evaluated the therapeutic effects of oral nano-curcumin in OLP. Kia’s randomized clinical trial involved 60 patients with erosive and atrophic OLP forms. The patients were divided into two groups, one treated with nano-curcumin and the other with prednisolone, for four weeks. Both groups showed a significant reduction in pain, burning sensation, and lesion size, with no significant differences between them. The results suggest that oral nano-curcumin could be an effective alternative for patients unable to tolerate corticosteroids or requiring cautious use. Further research, including long-term follow-ups to assess recurrence rates, is recommended to establish nano-curcumin as a new therapeutic option for OLP [5].

Currently, the most common treatment of OLP is based on systemic and/or topical corticosteroids but has limited side effects. Turmeric, known for its anti-inflammatory properties, contains curcuminoids, including curcumin, which has been studied for its beneficial properties. A 2007 randomized, double-blind, placebo-controlled study by N Chainani-Wu evaluated the efficacy and safety of curcuminoids as an adjunct to short-term corticosteroid therapy for the treatment of OLP. The patients were divided into two groups, one receiving prednisone and curcumin and the other prednisone and a placebo. The patients were evaluated for changes in symptoms and clinical signs for 7 weeks. Despite turmeric’s popularity as a traditional remedy, the study found no evident variations in the symptoms and indicators between the curcuminoid and placebo groups. The study was stopped early due to futility analysis, which showed that the probability of finding a notable distinction between the two groups was very low, even if the study had been completed with the full sample. The tolerability of curcuminoids was good, with few side effects reported [114].

### 4.6. The Potential Side Effects of Curcumin therapy

Curcumin, although known for its many health benefits, may also have some side effects that require attention. Among these, one of the most common is the possibility of causing stains on the mouth and teeth, especially following prolonged use or high doses. This phenomenon certainly has esthetic implications and can influence the patients’ acceptance of treatment. Stains can not only negatively affect the patient’s esthetic appearance, but also compromise confidence and self-esteem. This phenomenon, although it does not pose health risks, could however cause psychological and social distress to patients, requiring appropriate management during treatment [115]. A possible way to avoid this side effect is to use tetrahydrocurcumin, one of the main metabolites of curcumin. This substance does not possess the α,β-unsaturated carbonyl group and is white, which makes it lack the intense yellow pigment characteristic of curcumin. As a result, tetrahydrocurcumin has no risk of staining the teeth, offering a more esthetically pleasing option for people who want to take advantage of turmeric’s medicinal benefits without sacrificing the appearance of their teeth [116]. Therefore, it is essential that dental professionals inform patients fully and are transparent about the potential side effects of curcumin, thus ensuring an informed decision and effective management of esthetic expectations during the therapeutic journey. Furthermore, some individuals may experience allergic reactions to curcumin, which can range from mild skin irritations to more serious symptoms, such as facial swelling or difficulty breathing. It is therefore essential to conduct a careful risk–benefit assessment before using curcumin in dental treatments, especially in patients with a history of allergies or skin sensitivities [117]. It is also important to keep in mind that curcumin may interact with some medications; therefore, it is advisable to consult a healthcare professional before starting any therapy involving the use of this compound [115].

## 5. Conclusions

Considering the possibility of using new techniques and resources to solve medical issues, it has become more and more important to understand the benefits that nature provides. Curcumin is a prominent natural compound that has been extensively studied in the recent literature due to its anti-inflammatory, antioxidant, antibacterial, and anticancer activities. As a matter of fact, it shows promise as an adjuvant therapeutic agent for the treatment of several oral health issues, possibly providing more effective and safer alternatives to traditional medicines. It seems to be a very promising natural therapeutic agent in the treatment of oral cancer, mucositis, periodontal disease, and perhaps malignant illnesses. While curcumin offers numerous health benefits, its potential side effects, such as staining of the mouth and teeth, necessitate careful consideration. These esthetic concerns not only impact the patient’s appearance, but also their confidence and self-esteem, highlighting the importance of informed patient communication and proactive management during treatment. Additionally, allergic reactions and drug interactions underscore the need for thorough risk–benefit assessment and consultation with healthcare professionals before initiating curcumin-based therapies.

However, more research is required because its effectiveness varies depending on the condition and route of administration.

## Figures and Tables

**Table 1:** Summary of the selected articles.

Authors	Type of the Study	Age and Numbers of Participants	Aim of the Study	Materials and Clinical Data	Results and Percentages
Cindy Grace Pérez-Pacheco et al., 2021 [76]	Double-blind split-mouth randomized clinical trial.	Twenty male and female participants in good health, aged 30 years or older.	Examination of a single local application of nanoparticles loaded with curcumin as a complementary treatment for nonsurgical periodontal therapy to SRP.	SRP + empty nanoparticles or SRP + PLGA(Poly lactide-co-glicolide)/PLA(polu lactide) nanoparticles loaded with 50 μg of curcumin (N-Curcumin) were administered to twenty healthy patients with periodontitis. Monitoring was placed at baseline, 30, 90, and 180 days for PPD, CAL, and BOP.	In the periodontally infected areas, a single local dosage of nanoencapsulated curcumin did not enhance the clinical outcome.
Hayder Thabit Farhood et al., 2020 [77]	Clinical random split-mouth interventional study.	Twenty Iraqi patients aged 21–45 years, comprising 9 males and 11 females.	The purpose of the study was to evaluate the efficacy of subgingival SRP with a curcumin oral gel as an adjuvant treatment and to compare the results with SRP alone.	Patients with bilateral 5 to 7 mm pockets exclusively in the upper jaw and periodontitis (stage II/III, grade AC) were chosen at random. Using the split-mouth approach, one side was designated as the control group and received SRP alone; the other side was designated as the test group and received two applications of the curcumin oral gel in addition to SRP spaced one week apart.	According to the study’s findings, the curcumin oral gel significantly improved all clinical periodontal parameters when compared to the baseline, with the test group experiencing a more noticeable improvement in PPD, CAL, PI, and GI.
Mansour Al-Askar et al., 2022 [78]	Clinical study.	Two groups of seventy-six periodontitis patients were randomly assigned. The test and control groups’ mean ages were 57.2 ± 5.2 and 58.4 ± 7.3 years, respectively.	To evaluate the comparative analgesic effectiveness of mefenamic acid (MA) and curcumin administered orally following surgical periodontal treatment (SPT).	Patients were administered 200 mg curcumin capsules in the test group and 500 mg of MA in the control group.	Curcumin was not as effective as MA for treating pain and discomfort following SPT.
Reham Abdel-Fatah et al., 2023 [79]	Clinical study.	In the study, eighteen healthy volunteers and thirty-six periodontitis patients took part. The participants ranged in age from thirty to fifty-five years old, representing both genders.	To assess the impact of SRP in conjunction with a curcumin gel on salivary procalcitonin during treating periodontitis.	Three groups of participants were created: group I was made up of people with healthy gums; group II had a scaling and root planing treatment; and group III received weekly applications of a curcumin gel for four weeks following SRP treatment.	Compared to SRP, the application of the curcumin gel was observed to have a substantial impact on all clinical indicators.
Sanjeela Rakshith Guru et al., 2020 [80]	Clinical trial.	Forty-five people with long-term periodontitis, aged 25 to 50 years.	To compare the effectiveness of a 1% chlorhexidine gel and 2% curcumin with nanocarriers as a local drug delivery for the treatment of periodontal pockets.	Patients who had at least two teeth with pockets that were 5–7 mm deep were chosen. Microbiological analysis and clinical parameter assessment were performed at baseline, day 21, and day 45.	The findings demonstrated that all clinical parameters improved when the two local drug delivery (LDD) medications were administered in addition to SRP for chronic periodontitis.
Mallapragada Siddharth et al., 2020 [81]	Randomized controlled trial.	Twenty-five people in the age category of ≥30 years who were in general good health.	To evaluate the benefits of adding a 0.2% chlorhexidine gel and a 2% curcumin gel subgingivally as an adjuvant to scaling and root planing (SRP).	Subgingival administration of 0.2% chlorhexidine gel in the control sites and 2% curcumin gel in the experimental sites was carried out following a full-mouth SRP. Subgingival plaque samples were taken once more at one and three months, and measurements of site-specific periodontal parameters were made.	Periodontal measurements improved significantly according to statistical analysis in the experimental group (2% curcumin gel).
Sarah Adnan Alsalim et al., 2024 [82]	Clinical study.	Thirty-one head and neck cancer (HNC) patients over 16 years of age.	Comparison of the effectiveness of a curcumin oral gel versus the magic solution (mouthwash containing dexamethasone) for the treatment of radiation-induced oral mucositis (RIOM).	Thirty-one HNC patients were divided into those treated with the curcumin oral gel and those treated with the magic solution. Salivary epidermal growth factor (EGF) was quantified and RIOM was assessed using the WHO scale and visual analog scale (VAS).	During the radiation treatments, the patients who took the oral curcumin gel had increased salivary EGF levels and less severe RIOM.
Swikant Shah et al., 2020 [83]	Randomized controlled trial.	Seventy-four patients.	Analysis of the effects on RIOM in 74 patients with head and neck cancer who were set to receive an RT of 0.15% benzydamine mouthwash and 0.1% freshly produced curcumin utilizing nanoparticles.	Every week for six weeks, RIOM was evaluated once a week using the WHO criteria. Two types of analyses, namely modified intention to treat (MIT) and per protocol (PP), were conducted to test the null hypothesis that there is comparable effectiveness between the prevention and severity of RIOM.	While mouthwashes were not able to stop RIOM from happening altogether or lessen its severity, using a mouthwash containing 0.1% curcumin was able to greatly postpone the onset of RIOM.
Lindsay Boven MD et al., 2018 [64]	Clinical trial	Ten healthy patients, 2 female and 8 male, between the ages of 30 and 68 years.	Validation of the effectiveness of curcumin gum by measuring its effects and evaluating its release and transmucosal absorption.	The residual curcumin chewed gum, serum, and saliva were measured by comparing the two chewing protocols, the initial and the revised chewing. Using a multiplex analysis, the levels of 15 proinflammatory cytokines in serum were examined.	It seems that improving mucosal contact is essential for enhancing curcumin absorption and release.
Rita de Cássia Dias Viana Andrade et al., 2022 [84]	Comparative randomized trial study.	Thirty patients (over 18 years old).	Assessment of the impact of curcumin and blue LED-mediated antimicrobial photodynamic therapy (aPDT) and photobiomodulation (PBM) as an adjuvant treatment for oral mucositis undergoing chemotherapy or radiation therapy.	Thirty patients (above the age of eighteen years) undergoing radiation and/or chemotherapy for stable oral mucosal lesions. Patients were assigned to three groups: aPDT group, PBM group, and control group.	Mucositis and pain degree scores decreased in both the PBM and aPDT groups; however, the aPDT group demonstrated early clinical improvement in contrast to the PBM group and the control group. Regarding antibacterial efficacy, aPDT showed a greater reduction in Candida yeasts throughout the parameters that were studied.
Basudev Mahato et al., 2019 [85]	Clinical study.	Forty patients. Mean age of 34.75 years. Age range of 18–62 years.	Evaluation of the efficacy of curcumin, lycopene, and piperine as a combination in the management of OSMF.	Biocumin (Biochem India) tablet with Curcumin (500 mg), piperine (5 mg), and lycopene (25 mg) twice a day for 3 months. Clinical evaluations of the respondents were conducted every 15 days until a 3-month follow-up. Mouth opening (MO), mucosal flexibility (MF), tongue protrusion (TP), and burning sensation score on the VAS were the parameters.	A considerable enhancement in the VAS score for burning sensation and a rise in MO (*p* < 0.001), MF, and TP were noted. Reepithelialization was also seen in the post-treatment histological assessment. Col1A1-based immunohistochemical investigations revealed a reduction in collagen deposition.
Tej Prakash Soni et al., 2021 [86]	Randomized double-blind placebo-controlled trial.	Sixty patients with oral cancer aged between 18 and 70 years.	Examination the impact of a turmeric formulation on patients undergoing chemotherapy and radiation therapy for oral cancer in terms of oral mucositis.	For six weeks, chemoradiotherapy was administered in addition to the daily administration of a bio-enhanced turmeric formulation (BTF) capsules (low dose [1 g/day] or high dose [1.5 g/day]) or placebo.	Patients with oral cancer who experience severe oral mucositis, dysphagia, oral pain, and dermatitis due to chemotherapy and radiation therapy can greatly benefit from BTF (BCM-95^®^).
Mellekatte C Neetha et al., 2020 [87]	Double-blind, randomized preliminary study.	Sixty subjects with oral potentially malignant disorders (OPMDs)	Evaluation of the combined effects of curcumin and a green tea extract in patients with OPMDs and determination of these chemopreventive drugs’ mode of action by evaluating the relevant biomarkers.	For three months, the patients were randomized to receive either a green tea extract (800 mg/day applied topically and systemically) or curcumin (950 mg/day applied topically and systemically) or a combination therapy. There were 20 patients in each group. Biomarkers (p53, cyclin D1, and Ki67) were assessed in biopsies taken at baseline and after 12 weeks.	When comparing the combination group to the curcumin and green tea extract groups, the combination group had a greater clinical response rate. When comparing the combination group’s p53, Ki67, and cyclin D1 expression after three months to the baseline, there was a statistically significant downregulation.
Saroj K Basak et al., 2020 [88]	Double-blind, randomized, placebo-controlled, phase 1 clinical trial.	Thirteen normal subjects and twelve patients with oral cancer.	To assess that APG-157 could serve as a therapeutic drug in combination with immunotherapy.	Two doses, either 100 mg or 200 mg, were administered every hour for a total of 3 h. Blood and saliva samples were collected before treatment and at 1 h, 2 h, 3 h, and 24 h post-treatment.	The results indicated that circulating concentrations of curcumin and its analogs peaked at 3 h, accompanied by decreased concentrations of interleukin 1β (Il-1β), interleukin- 6 (Il-6), and interleukin-8 (Il-8) in the salivary supernatant fluid of the cancer patients.
Deshmukh and Bagewadi, 2014 [89]	Randomized clinical trial.	Sixty patients of either sex.	To assess and compare the efficacy of a curcumin gel with triamcinolone acetonide gel in the treatment of minor RAS.	The patients were randomly assigned to two groups: the curcumin gel group (Group I) and the triamcinolone acetonide gel group (Group II). Patients applied the gel three times a day to each ulcer. The evaluation of effectiveness was based on the time needed for pain, size, and number of ulcers to regress.	Both groups showed a significant improvement in the size, pain, number, and duration of the ulcers within 7 days. No significant difference between the two groups in the treatment of recurrent aphthous stomatitis (RAS) were found.
Adhikari et al., 2022 [90]	Randomized, double-blind, parallel design, clinical trial.	Thirty-four patients.	To determine the efficacy of curcumin in combination with intralesional dexamethasone with hyaluronidase in the treatment of OSMF.	For six weeks, the patients were first treated with intralesional dexamethasone and hyaluronidase. Following this, Group A received curcumin (2 gm/day), while Group B received a placebo. At baseline and during the follow-up visits at 6, 8, and 12 weeks, measurements of interincisal mouth openness, TP, cheek flexibility, and VAS grading for burning sensation of the oral mucosa were taken.	Both groups demonstrated significant improvements in MO, cheek flexibility, TP, and oral mucosa burning sensation. In Group A, the mean differences in MO at 6, 8, and 12 weeks were 8.82 mm, 8.71 mm, and 8.06 mm, compared to Group B‘s mean differences of 5.53 mm, 5.35 mm, and 4.94 mm, respectively. Similar trends were observed in cheek flexibility and TP. Both groups achieved 100% improvement in burning sensation at all follow-up intervals.
Kia et al., 2020 [91]	Randomized controlled trial.	Sixty patients.	To evaluate the therapeutic effect of oral nano-curcumin as an alternative treatment for oral lichen planus (OLP) compared to prednisolone.	Patients were divided into two groups receiving either 80 mg nano-curcumin or 10 mg prednisolone treatments for 1 month. Pain severity and burning sensation were analyzed using the VAS scale, while lesion size was assessed using the Thongprasom scale.	Both the nano-curcumin and prednisolone groups showed a decrease in pain, burning sensation, and OLP lesions, with no statistically significant difference between the two groups.

## Data Availability

All of the data is contained within the article.

## Abbreviations

aPDT	Antimicrobial photodynamic therapy
BOP	Bleeding on probing
BTF	Bio-enhanced turmeric formulation
CAL	Clinical attachment level
CMS	Carboxymethyl starch
COX-2	Cyclooxygenase-2
CS	Chitosan
CRP	C-reactive protein
DNA	Deoxyribonucleic acid
EGF	Epidermal growth factor
EMT	Epithelial–mesenchymal transition
GI	Gingival index
HNC	Head and neck cancer
IL-1β	Interleukin 1β
IL-6	Interleukin-6
IL-8	Interleukin-8
LDD	Local drug delivery
MA	Mefenamic acid
MDA	Malondialdehyde
MDSC	Myeloid-derived suppressor cells
MF	Mucosal flexibility
MIT	Modified intention to treat
MMT	Montmorillonite
MO	Mouth opening
MOMI	Modified oral mucositis index
NF- κB	Nuclear factor kappa B
NRS	Numerical rating scale
OLP	Oral lichen planus
OPMDs	Oral potentially malignant disorders
OSCC	Oral squamous carcinoma
OSMF	Oral submucosal fibrosis
PBM	Photobiomodulation
PCT	Procalcitonin
PI	Plaque index
PLA	Poly lactide
PLGA	Poly lactide-co-glicolide
PP	Per protocol
PPD	Periodontal probing depth
RANK	Receptor activator of nuclear factor κB
RANKL	Receptor activator of nuclear factor κΒ ligand
RAS	Recurrent aphthous stomatitis
RIOM	Radiation-induced oral mucositis
ROS	Reactive oxygen species
PLGA	Poly lactide-co-glicolide
SRP	Scaling and root planing
SPT	Surgical periodontal therapy
TGF-β	Transforming growth factor β
TP	Tongue protrusion
VAS	Visual analog scale
8-OHdG	8-hydroxydeoxyguanosine
